# 2-(4-Isopropyl­benzylidene)propanoic acid

**DOI:** 10.1107/S1600536808020801

**Published:** 2008-07-12

**Authors:** Niaz Muhammad, M. Nawaz Tahir, Saqib Ali, Zia-ur-Rehman, Muhammad Akram Kashmiri

**Affiliations:** a Department of Chemistry, Quaid-i-Azam University, Islamabad 45320, Pakistan; b Department of Physics, University of Sargodha, Sagrodha, Pakistan; c Department of Chemistry, Government College University, Lahore, Pakistan

## Abstract

The two mol­ecules in the asymmetric unit of the title compound, C_13_H_16_O_2_, form dimers through O—H⋯O hydrogen bonding, resulting in *R*_2_^2^(8) rings. Each carboxyl­ O atom is involved in inter­amolecular C—H⋯O hydrogen bonds, forming five-membered rings. There exist dissimilar dihedral angles within the two mol­ecules, for example the carboxylate and isopropyl groups make dihedral angles of 59.6 (4) and 71.7 (3)° in the two molecules. There are no intermolecular π inter­actions.

## Related literature

For related literature, see: Burt (2004 ▶); Hertog *et al.* (1995 ▶); Ma & Hayes (2004 ▶); Muhammad *et al.* (2007 ▶).
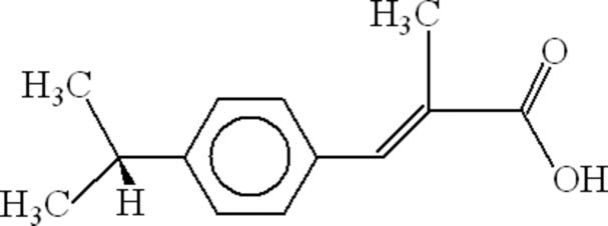


## Experimental

### 

#### Crystal data

C_13_H_16_O_2_
*M* *_r_* = 204.26
Triclinic, 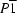

*a* = 9.8406 (4) Å
*b* = 10.5739 (4) Å
*c* = 11.9142 (5) Å
α = 96.330 (2)°
β = 98.486 (3)°
γ = 104.497 (2)°
*V* = 1172.99 (8) Å^3^
*Z* = 4
Mo *K*α radiation radiation
μ = 0.08 mm^−1^
*T* = 296 (2) K
0.30 × 0.18 × 0.12 mm

#### Data collection

Bruker Kappa APEXII CCD diffractometer
Absorption correction: multi-scan (*SADABS*; Bruker, 2005 ▶) *T*_min_ = 0.977, *T*_max_ = 0.986
23066 measured reflections
6000 independent reflections
2803 reflections with *I* > 3σ(*I*)
*R*_int_ = 0.033

#### Refinement

*R*[*F* ^2^ > 2σ(*F* ^2^)] = 0.053
*wR*(*F* ^2^) = 0.157
*S* = 1.04
6000 reflections
290 parameters
H atoms treated by a mixture of independent and constrained refinement
Δρ_max_ = 0.28 e Å^−3^
Δρ_min_ = −0.23 e Å^−3^

### 

Data collection: *APEX2* (Bruker, 2007 ▶); cell refinement: *APEX2*; data reduction: *SAINT* (Bruker, 2007 ▶); program(s) used to solve structure: *SHELXS97* (Sheldrick, 2008 ▶); program(s) used to refine structure: *SHELXL97* (Sheldrick, 2008 ▶); molecular graphics: *ORTEP-3 for Windows* (Farrugia, 1997 ▶) and *PLATON* (Spek, 2003 ▶); software used to prepare material for publication: *WinGX* (Farrugia, 1999 ▶) and *PLATON*.

## Supplementary Material

Crystal structure: contains datablocks global, I. DOI: 10.1107/S1600536808020801/jh2064sup1.cif

Structure factors: contains datablocks I. DOI: 10.1107/S1600536808020801/jh2064Isup2.hkl

Additional supplementary materials:  crystallographic information; 3D view; checkCIF report

## Figures and Tables

**Table 1:** Hydrogen-bond geometry (Å, °)

*D*—H⋯*A*	*D*—H	H⋯*A*	*D*⋯*A*	*D*—H⋯*A*
O1—H1⋯O4^i^	0.94 (4)	1.71 (4)	2.644 (4)	175 (3)
O3—H3*A*⋯O2^ii^	0.93 (4)	1.71 (4)	2.631 (3)	169 (3)
C3—H3⋯O1	0.96 (3)	2.35 (2)	2.707 (4)	101.2 (16)
C13—H13*A*⋯O2	0.96	2.28	2.759 (4)	110
C16—H16⋯O3	0.91 (3)	2.31 (2)	2.698 (4)	105.1 (18)
C26—H26*A*⋯O4	0.96	2.30	2.770 (4)	110

Symmetry codes: (i) 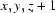; (ii) 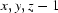.

**Fig. 1.**
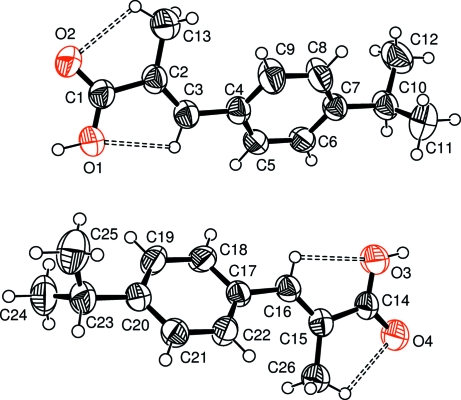
ORTEP drawing of the title compound, (C13H16O2)2 with the atom numbering scheme. The thermal ellipsoids are drawn at the 50% probability level. H-atoms are shown by small circles of arbitrary radii. The intramolecular H-bonds are shown by doted lines.

**Fig. 2.**
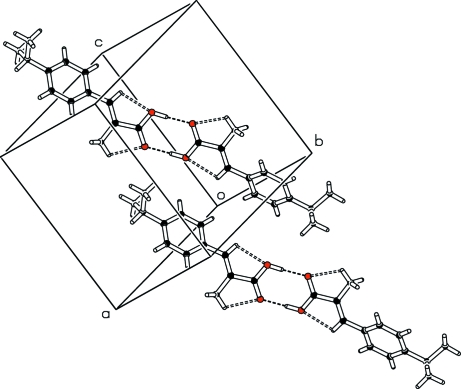
The partial packing figure (PLATON: Spek, 2003) which shows the dimeric nature of the compound.

## supplementary crystallographic information

### Comment

Cinnamic acids and their derivatives are widely used chemicals in a variety of fields (Ma & Hayes, 2004). They posses antibacterial, antifungal and parasite fighting abilities (Burt, 2004). A derivative of cinnamic acid is an important pharmaceutical for high blood pressure and stroke prevention and possess antitumour activity (Hertog *et al.*, 1995).

The crystal structure of 3-(4-Bromophenyl)-2-methylacrylic acid (Muhammad *et al.*, 2007) has been reported. The title compound (I) have a replacement of Br-atom with isopropyl at the same position. The ligand has been prepared to synthesize various organotin complexes.

The crystallographic asymmetric unit consists of two ligands. These two ligands form dimers through O—H···O hydrogen bonding with each other by completing a *R*_2_^2^(8) rings (Table 1, Fig 2). Each O-atom of carboxylate ligands is involved in interamolecular H-bonds of C—H···O type forming five-membered rings. The bond distances in the benzene ring (C4—C9) have values in the range 1.372 (4)–1.390 (4) Å, whereas in (C17—C22) its range is 1.374 (4)–1.384 (4) Å. The bond angle (C11—C10—C12) of isopropyl moiety is 111.2 (3)°, whereas the same for (C24—C23—C25) is 109.8 (3)°. There exist a dissimilar dihedral angles within the two moieties. The dihedral angles of the isopropyl moieties (C10/C11/C12) and (C23/C24/C25) with their adjacent benzene rings (C4—C9) and (C17—C22) have values of 86.80 (13)° and 85.06 (13)°, respectively. The dihedral angles of the moieties (C2/C3/C13) and (C15/C16/C26) with their adjacent carboxylate moities (C1/O1/O2) and (C14/O3/O4) have values of 7.65 (51)° and 7.06 (43)°, respectively, whereas with benzene rings (C4—C9) and (C17—C22), the values of their dihedral angles is 32.18 (23)° and 34.49 (20)°, respectively. The dihedral angle between the benzene rings of two ligands is 83.34 (9)°. There does not exist any kind of π-interaction.

### Experimental

Compound (I) was prepared according to the reported procedure in literature (Muhammad *et al.*, 2007). A mixture of 4-isopropylbenzaldehyde (10 mmol, 1.51 ml), methylmalonic acid (2.36 g, 20 mmol) and piperidine (20 mmol, 1.98 ml) in pyridine (12.5 ml) solution was heated on a steam-bath for 24 h. The reaction mixture was cooled and added to a mixture of 25 ml of concentrated HCl and 50 g of ice. The precipitate formed in the acidified mixture was filtered off and washed with ice-cold water. The product was recrystallized from ethanol. The yield was 80%.

### Refinement

H atoms were positioned geometrically, with C—H = 0.93, and 0.96 Å for aromatic and methyl H, and constrained to ride on their parent atoms, while the coordinates of all other H-atoms were refined. The H-atoms were treated as isotropic with U_iso_(H) = xU_eq_(C,O), where x = 1.5 for methyl H, and x = 1.2 for all other H atoms.

### Crystal data

**Table:**

C_13_H_16_O_2_	*Z* = 4
*M**_r_* = 204.26	*F*_000_ = 440
Triclinic, *P*1	*D*_x_ = 1.157 Mg m^−^^3^
Hall symbol: -P 1	Mo *K*α radiation radiation λ = 0.71073 Å
*a* = 9.8406 (4) Å	Cell parameters from 2803 reflections
*b* = 10.5739 (4) Å	θ = 2.2–28.8º
*c* = 11.9142 (5) Å	µ = 0.08 mm^−^^1^
α = 96.330 (2)º	*T* = 296 (2) K
β = 98.486 (3)º	Prismatic, colourless
γ = 104.497 (2)º	0.30 × 0.18 × 0.12 mm
*V* = 1172.99 (8) Å^3^	

### Data collection

**Table:**

Bruker Kappa APEXII CCD diffractometer	6000 independent reflections
Radiation source: fine-focus sealed tube	2803 reflections with *I* > 3σ(*I*)
Monochromator: graphite	*R*_int_ = 0.033
Detector resolution: 7.4 pixels mm^-1^	θ_max_ = 28.8º
*T* = 296(2) K	θ_min_ = 2.2º
ω scans	*h* = −13→13
Absorption correction: multi-scan(SADABS; Bruker, 2005)	*k* = −13→14
*T*_min_ = 0.977, *T*_max_ = 0.986	*l* = −15→16
23066 measured reflections	

### Refinement

**Table:**

Refinement on *F*^2^	Hydrogen site location: inferred from neighbouring sites
Least-squares matrix: full	H atoms treated by a mixture of independent and constrained refinement
*R*[*F*^2^ > 2σ(*F*^2^)] = 0.053	*w* = 1/[σ^2^(*F*_o_^2^) + (0.0998*P*)^2^ + 0.3929*P*] where *P* = (*F*_o_^2^ + 2*F*_c_^2^)/3
*wR*(*F*^2^) = 0.157	(Δ/σ)_max_ = 0.002
*S* = 1.04	Δρ_max_ = 0.28 e Å^−^^3^
6000 reflections	Δρ_min_ = −0.23 e Å^−^^3^
290 parameters	Extinction correction: empirical, Fc^*^=kFc[1+0.001xFc^2^λ^3^/sin(2θ)]^-1/4^
Primary atom site location: structure-invariant direct methods	Extinction coefficient: 0.020 (3)
Secondary atom site location: difference Fourier map	

### Special details

**Table:**

Geometry. Bond distances, angles *etc*. have been calculated using the rounded fractional coordinates. All su's are estimated from the variances of the (full) variance-covariance matrix. The cell e.s.d.'s are taken into account in the estimation of distances, angles and torsion angles
Refinement. Refinement of *F*^2^ against ALL reflections. The weighted *R*-factor *wR* and goodness of fit *S* are based on *F*^2^, conventional *R*-factors *R* are based on *F*, with *F* set to zero for negative *F*^2^. The threshold expression of *F*^2^ > σ(*F*^2^) is used only for calculating *R*-factors(gt) *etc*. and is not relevant to the choice of reflections for refinement. *R*-factors based on *F*^2^ are statistically about twice as large as those based on *F*, and *R*- factors based on ALL data will be even larger.

### Fractional atomic coordinates and isotropic or equivalent isotropic displacement parameters (Å^2^)

**Table:**

	*x*	*y*	*z*	*U*_iso_*/*U*_eq_	
O1	0.4946 (3)	0.6966 (3)	0.41038 (19)	0.0929 (11)	
O2	0.3929 (3)	0.8025 (3)	0.52698 (18)	0.0859 (10)	
C1	0.4167 (3)	0.7736 (3)	0.4302 (3)	0.0604 (10)	
C2	0.3538 (3)	0.8277 (3)	0.3319 (2)	0.0558 (10)	
C3	0.3665 (3)	0.7807 (3)	0.2271 (2)	0.0572 (10)	
C4	0.3150 (3)	0.8149 (3)	0.1158 (2)	0.0529 (9)	
C5	0.2805 (3)	0.7198 (3)	0.0194 (2)	0.0567 (10)	
C6	0.2312 (3)	0.7457 (3)	−0.0873 (2)	0.0575 (9)	
C7	0.2160 (3)	0.8681 (3)	−0.1039 (2)	0.0536 (9)	
C8	0.2534 (4)	0.9643 (3)	−0.0083 (3)	0.0816 (13)	
C9	0.3021 (4)	0.9397 (3)	0.0992 (3)	0.0802 (13)	
C10	0.1631 (3)	0.8967 (3)	−0.2215 (3)	0.0652 (11)	
C11	0.2802 (4)	0.9901 (4)	−0.2652 (3)	0.0981 (16)	
C12	0.0319 (4)	0.9478 (4)	−0.2243 (3)	0.0919 (17)	
C13	0.2769 (4)	0.9280 (3)	0.3617 (3)	0.0818 (12)	
O3	0.5215 (3)	0.7354 (3)	−0.28826 (18)	0.0829 (9)	
O4	0.6264 (3)	0.6312 (3)	−0.40400 (18)	0.0887 (10)	
C14	0.6113 (3)	0.6697 (3)	−0.3062 (2)	0.0559 (10)	
C15	0.6987 (3)	0.6413 (3)	−0.2046 (2)	0.0531 (9)	
C16	0.6664 (3)	0.6713 (3)	−0.1024 (2)	0.0538 (9)	
C17	0.7335 (3)	0.6564 (2)	0.0123 (2)	0.0490 (9)	
C18	0.6479 (3)	0.6330 (3)	0.0942 (2)	0.0640 (10)	
C19	0.7024 (3)	0.6191 (3)	0.2041 (2)	0.0697 (13)	
C20	0.8461 (3)	0.6321 (3)	0.2382 (2)	0.0548 (9)	
C21	0.9313 (3)	0.6562 (3)	0.1571 (2)	0.0561 (9)	
C22	0.8773 (3)	0.6688 (3)	0.0464 (2)	0.0562 (9)	
C23	0.9076 (4)	0.6231 (3)	0.3602 (3)	0.0708 (11)	
C24	0.8264 (4)	0.5008 (4)	0.4022 (3)	0.0926 (16)	
C25	0.9114 (4)	0.7468 (4)	0.4414 (3)	0.0945 (16)	
C26	0.8123 (3)	0.5764 (3)	−0.2296 (3)	0.0682 (11)	
H1	0.537 (4)	0.669 (4)	0.476 (3)	0.1113*	
H3	0.410 (3)	0.709 (3)	0.218 (2)	0.0686*	
H5	0.29082	0.63593	0.02693	0.0680*	
H6	0.20748	0.67847	−0.14988	0.0690*	
H8	0.24547	1.04865	−0.01676	0.0975*	
H9	0.32671	1.00736	0.16152	0.0961*	
H10	0.140 (3)	0.809 (3)	−0.279 (3)	0.0784*	
H11A	0.24320	1.00662	−0.33965	0.1470*	
H11B	0.35749	0.95085	−0.27047	0.1470*	
H11C	0.31399	1.07189	−0.21301	0.1470*	
H12A	0.00214	0.96492	−0.30028	0.1381*	
H12B	0.05422	1.02807	−0.17077	0.1381*	
H12C	−0.04376	0.88278	−0.20377	0.1381*	
H13A	0.28465	0.94509	0.44353	0.1227*	
H13B	0.17804	0.89513	0.32631	0.1227*	
H13C	0.31834	1.00840	0.33437	0.1227*	
H3A	0.470 (4)	0.748 (3)	−0.356 (3)	0.0995*	
H16	0.587 (3)	0.701 (3)	−0.102 (2)	0.0645*	
H18	0.55108	0.62637	0.07461	0.0768*	
H19	0.64129	0.60065	0.25632	0.0836*	
H21	1.02841	0.66423	0.17740	0.0674*	
H22	0.93853	0.68585	−0.00598	0.0674*	
H23	1.009 (4)	0.624 (3)	0.362 (3)	0.0851*	
H24A	0.86976	0.49923	0.47953	0.1391*	
H24B	0.82917	0.42321	0.35347	0.1391*	
H24C	0.72907	0.50259	0.40018	0.1391*	
H25A	0.95096	0.73954	0.51830	0.1420*	
H25B	0.81621	0.75538	0.43888	0.1420*	
H25C	0.96945	0.82322	0.41810	0.1420*	
H26A	0.81376	0.56746	−0.31044	0.1023*	
H26B	0.79248	0.49055	−0.20633	0.1023*	
H26C	0.90349	0.62961	−0.18800	0.1023*	

### Atomic displacement parameters (Å^2^)

**Table:**

	*U*^11^	*U*^22^	*U*^33^	*U*^12^	*U*^13^	*U*^23^
O1	0.124 (2)	0.133 (2)	0.0530 (14)	0.0852 (18)	0.0161 (13)	0.0295 (13)
O2	0.1026 (17)	0.129 (2)	0.0474 (13)	0.0638 (15)	0.0173 (12)	0.0229 (12)
C1	0.0587 (17)	0.0766 (19)	0.0484 (18)	0.0235 (14)	0.0068 (13)	0.0122 (14)
C2	0.0520 (16)	0.0657 (17)	0.0501 (17)	0.0173 (13)	0.0042 (12)	0.0140 (13)
C3	0.0579 (17)	0.0641 (17)	0.0521 (18)	0.0204 (14)	0.0070 (13)	0.0150 (13)
C4	0.0521 (15)	0.0582 (16)	0.0479 (16)	0.0154 (12)	0.0041 (12)	0.0113 (12)
C5	0.0657 (17)	0.0561 (16)	0.0539 (18)	0.0277 (13)	0.0077 (13)	0.0108 (13)
C6	0.0669 (17)	0.0578 (16)	0.0485 (16)	0.0256 (13)	0.0022 (13)	0.0015 (12)
C7	0.0585 (16)	0.0545 (15)	0.0463 (16)	0.0175 (12)	0.0013 (12)	0.0073 (12)
C8	0.132 (3)	0.0526 (17)	0.059 (2)	0.0344 (18)	−0.0045 (19)	0.0109 (14)
C9	0.127 (3)	0.0553 (17)	0.0504 (19)	0.0250 (17)	−0.0045 (18)	0.0022 (13)
C10	0.082 (2)	0.0626 (18)	0.0511 (18)	0.0279 (16)	−0.0016 (15)	0.0081 (14)
C11	0.119 (3)	0.114 (3)	0.070 (2)	0.033 (2)	0.028 (2)	0.033 (2)
C12	0.096 (3)	0.101 (3)	0.086 (3)	0.050 (2)	−0.006 (2)	0.021 (2)
C13	0.096 (2)	0.100 (2)	0.060 (2)	0.051 (2)	0.0051 (17)	0.0127 (17)
O3	0.0924 (16)	0.1221 (19)	0.0495 (13)	0.0610 (15)	0.0026 (11)	0.0178 (12)
O4	0.121 (2)	0.1225 (19)	0.0434 (13)	0.0694 (16)	0.0134 (12)	0.0186 (12)
C14	0.0642 (17)	0.0628 (16)	0.0431 (17)	0.0216 (14)	0.0066 (13)	0.0128 (12)
C15	0.0584 (16)	0.0521 (14)	0.0479 (16)	0.0146 (12)	0.0059 (12)	0.0105 (12)
C16	0.0567 (16)	0.0578 (16)	0.0483 (17)	0.0213 (13)	0.0034 (13)	0.0093 (12)
C17	0.0559 (16)	0.0495 (14)	0.0431 (15)	0.0198 (11)	0.0046 (12)	0.0069 (11)
C18	0.0521 (16)	0.098 (2)	0.0483 (17)	0.0340 (15)	0.0073 (13)	0.0095 (15)
C19	0.0619 (19)	0.113 (3)	0.0445 (17)	0.0346 (17)	0.0169 (14)	0.0206 (16)
C20	0.0565 (17)	0.0635 (16)	0.0466 (16)	0.0220 (13)	0.0048 (13)	0.0109 (12)
C21	0.0468 (15)	0.0680 (17)	0.0530 (17)	0.0167 (12)	0.0035 (13)	0.0116 (13)
C22	0.0537 (16)	0.0681 (17)	0.0469 (16)	0.0138 (13)	0.0112 (12)	0.0131 (13)
C23	0.0678 (19)	0.100 (2)	0.0496 (18)	0.0323 (18)	0.0030 (15)	0.0207 (16)
C24	0.132 (3)	0.093 (3)	0.057 (2)	0.038 (2)	0.008 (2)	0.0242 (18)
C25	0.110 (3)	0.099 (3)	0.057 (2)	0.010 (2)	−0.0022 (19)	0.0044 (18)
C26	0.080 (2)	0.077 (2)	0.0547 (19)	0.0349 (16)	0.0105 (15)	0.0105 (14)

### Geometric parameters (Å, °)

**Table:**

O1—C1	1.277 (4)	C13—H13A	0.9600
O2—C1	1.231 (4)	C13—H13B	0.9600
O1—H1	0.94 (4)	C13—H13C	0.9600
O3—C14	1.281 (4)	C14—C15	1.482 (4)
O4—C14	1.234 (3)	C15—C16	1.328 (4)
O3—H3A	0.93 (4)	C15—C26	1.499 (4)
C1—C2	1.481 (4)	C16—C17	1.470 (3)
C2—C13	1.491 (5)	C17—C18	1.384 (4)
C2—C3	1.327 (3)	C17—C22	1.382 (4)
C3—C4	1.465 (4)	C18—C19	1.378 (3)
C4—C5	1.380 (4)	C19—C20	1.380 (4)
C4—C9	1.390 (4)	C20—C21	1.374 (4)
C5—C6	1.374 (4)	C20—C23	1.513 (4)
C6—C7	1.372 (4)	C21—C22	1.381 (3)
C7—C10	1.511 (4)	C23—C24	1.518 (5)
C7—C8	1.379 (4)	C23—C25	1.528 (5)
C8—C9	1.376 (5)	C16—H16	0.91 (3)
C10—C11	1.515 (5)	C18—H18	0.9300
C10—C12	1.517 (5)	C19—H19	0.9300
C3—H3	0.96 (3)	C21—H21	0.9300
C5—H5	0.9300	C22—H22	0.9300
C6—H6	0.9300	C23—H23	0.99 (4)
C8—H8	0.9300	C24—H24A	0.9600
C9—H9	0.9300	C24—H24B	0.9600
C10—H10	1.04 (3)	C24—H24C	0.9600
C11—H11A	0.9600	C25—H25A	0.9600
C11—H11B	0.9600	C25—H25B	0.9600
C11—H11C	0.9600	C25—H25C	0.9600
C12—H12C	0.9600	C26—H26A	0.9600
C12—H12B	0.9600	C26—H26B	0.9600
C12—H12A	0.9600	C26—H26C	0.9600
			
O1···O4^i^	2.644 (4)	H5···C17^iv^	3.0200
O2···O3^i^	2.631 (3)	H6···H10	2.3100
O2···C14^i^	3.377 (4)	H6···H24B^iv^	2.4800
O3···O2^ii^	2.631 (3)	H8···H11C	2.5500
O4···C1^ii^	3.382 (4)	H8···H12B	2.3800
O4···O1^ii^	2.644 (4)	H8···C16^iii^	2.9900
O1···H19	2.7500	H8···C17^iii^	3.0700
O1···H3A^i^	2.83 (4)	H8···C12	2.9000
O1···H3	2.35 (2)	H8···C11	3.0400
O2···H11B^i^	2.8400	H9···C13	2.6900
O2···H13A	2.2800	H9···C2	2.9600
O2···H3A^i^	1.71 (4)	H9···O3^iii^	2.8800
O3···H9^iii^	2.8800	H9···H13C	2.0700
O3···H16	2.31 (2)	H10···H6	2.3100
O3···H1^ii^	2.86 (4)	H11A···H12A	2.4300
O4···H1^ii^	1.71 (4)	H11B···O2^ii^	2.8400
O4···H26A	2.3000	H11C···C8	2.8800
C1···O4^i^	3.382 (4)	H11C···H8	2.5500
C9···C13	3.187 (5)	H12A···H13B^v^	2.5900
C13···C9	3.187 (5)	H12A···H11A	2.4300
C14···O2^ii^	3.377 (4)	H12B···C8	2.8100
C22···C26	3.246 (4)	H12B···H8	2.3800
C26···C22	3.246 (4)	H13A···O2	2.2800
C1···H3A^i^	2.58 (3)	H13B···H25C^vi^	2.4600
C2···H9	2.9600	H13B···H12A^v^	2.5900
C3···H26B^iv^	2.8700	H13C···C9	2.7900
C5···H18	3.0700	H13C···H9	2.0700
C8···H11C	2.8800	H16···O3	2.31 (2)
C8···H12B	2.8100	H16···H18	2.3700
C9···H13C	2.7900	H18···C5	3.0700
C11···H8	3.0400	H18···H3	2.5700
C12···H8	2.9000	H18···H5	2.5700
C13···H9	2.6900	H18···H16	2.3700
C14···H1^ii^	2.59 (4)	H19···O1	2.7500
C15···H22	2.9900	H19···C24	2.8100
C16···H8^iii^	2.9900	H19···H24C	2.2700
C17···H5^iv^	3.0200	H21···H23	2.3100
C17···H8^iii^	3.0700	H22···C15	2.9900
C19···H24C	2.7700	H22···C26	2.7600
C19···H25B	2.9200	H22···H26C	2.1400
C22···H26C	2.8400	H23···H21	2.3100
C24···H19	2.8100	H24A···H25A	2.4300
C26···H22	2.7600	H24A···H24A^vii^	2.5300
H1···O4^i^	1.71 (4)	H24B···H6^iv^	2.4800
H1···O3^i^	2.86 (4)	H24C···C19	2.7700
H1···C14^i^	2.59 (4)	H24C···H19	2.2700
H1···H3A^i^	2.33 (5)	H24C···H25B	2.5600
H3···H5	2.3500	H25A···H24A	2.4300
H3···O1	2.35 (2)	H25B···C19	2.9200
H3···H18	2.5700	H25B···H24C	2.5600
H3···H26B^iv^	2.4900	H25C···H13B^viii^	2.4600
H3A···C1^ii^	2.58 (3)	H26A···O4	2.3000
H3A···O1^ii^	2.83 (4)	H26B···C3^iv^	2.8700
H3A···O2^ii^	1.71 (4)	H26B···H3^iv^	2.4900
H3A···H1^ii^	2.33 (5)	H26C···C22	2.8400
H5···H3	2.3500	H26C···H22	2.1400
H5···H18	2.5700		
			
C1—O1—H1	115 (2)	H13A—C13—H13C	109.00
C14—O3—H3A	113 (2)	O3—C14—O4	122.0 (3)
O1—C1—O2	122.1 (3)	O3—C14—C15	117.7 (2)
O1—C1—C2	117.7 (3)	O4—C14—C15	120.4 (3)
O2—C1—C2	120.2 (3)	C14—C15—C16	117.6 (3)
C1—C2—C13	115.4 (2)	C14—C15—C26	115.6 (2)
C1—C2—C3	118.2 (3)	C16—C15—C26	126.8 (3)
C3—C2—C13	126.4 (3)	C15—C16—C17	130.1 (3)
C2—C3—C4	129.9 (3)	C16—C17—C22	125.0 (2)
C3—C4—C9	124.2 (3)	C18—C17—C22	117.0 (2)
C3—C4—C5	119.0 (3)	C16—C17—C18	117.9 (3)
C5—C4—C9	116.7 (3)	C17—C18—C19	121.7 (3)
C4—C5—C6	121.8 (3)	C18—C19—C20	121.2 (3)
C5—C6—C7	121.7 (3)	C19—C20—C21	117.1 (2)
C8—C7—C10	121.6 (3)	C21—C20—C23	121.5 (3)
C6—C7—C8	116.7 (2)	C19—C20—C23	121.4 (3)
C6—C7—C10	121.6 (2)	C20—C21—C22	122.1 (3)
C7—C8—C9	122.2 (3)	C17—C22—C21	120.9 (3)
C4—C9—C8	120.8 (3)	C20—C23—C25	110.6 (3)
C7—C10—C12	112.2 (3)	C24—C23—C25	109.8 (3)
C7—C10—C11	111.5 (3)	C20—C23—C24	112.6 (3)
C11—C10—C12	111.2 (3)	C15—C16—H16	116.4 (15)
C2—C3—H3	118.7 (14)	C17—C16—H16	113.4 (15)
C4—C3—H3	111.3 (14)	C17—C18—H18	119.00
C6—C5—H5	119.00	C19—C18—H18	119.00
C4—C5—H5	119.00	C18—C19—H19	119.00
C5—C6—H6	119.00	C20—C19—H19	119.00
C7—C6—H6	119.00	C20—C21—H21	119.00
C9—C8—H8	119.00	C22—C21—H21	119.00
C7—C8—H8	119.00	C17—C22—H22	120.00
C4—C9—H9	120.00	C21—C22—H22	120.00
C8—C9—H9	120.00	C20—C23—H23	107 (2)
C11—C10—H10	103.8 (18)	C24—C23—H23	111.1 (19)
C12—C10—H10	110.3 (17)	C25—C23—H23	105.4 (19)
C7—C10—H10	107.5 (19)	C23—C24—H24A	109.00
C10—C11—H11B	109.00	C23—C24—H24B	109.00
C10—C11—H11C	109.00	C23—C24—H24C	109.00
H11A—C11—H11B	110.00	H24A—C24—H24B	109.00
H11A—C11—H11C	110.00	H24A—C24—H24C	109.00
H11B—C11—H11C	109.00	H24B—C24—H24C	109.00
C10—C11—H11A	109.00	C23—C25—H25A	109.00
C10—C12—H12A	109.00	C23—C25—H25B	109.00
C10—C12—H12C	109.00	C23—C25—H25C	109.00
H12A—C12—H12B	109.00	H25A—C25—H25B	109.00
H12A—C12—H12C	109.00	H25A—C25—H25C	109.00
H12B—C12—H12C	109.00	H25B—C25—H25C	109.00
C10—C12—H12B	110.00	C15—C26—H26A	109.00
C2—C13—H13A	110.00	C15—C26—H26B	109.00
C2—C13—H13C	109.00	C15—C26—H26C	109.00
H13A—C13—H13B	109.00	H26A—C26—H26B	109.00
C2—C13—H13B	109.00	H26A—C26—H26C	109.00
H13B—C13—H13C	109.00	H26B—C26—H26C	109.00
			
O1—C1—C2—C3	8.4 (5)	O3—C14—C15—C16	8.1 (4)
O1—C1—C2—C13	−173.7 (3)	O3—C14—C15—C26	−174.0 (3)
O2—C1—C2—C3	−171.3 (3)	O4—C14—C15—C16	−172.2 (3)
O2—C1—C2—C13	6.6 (5)	O4—C14—C15—C26	5.7 (4)
C1—C2—C3—C4	179.7 (3)	C14—C15—C16—C17	−179.3 (3)
C13—C2—C3—C4	2.0 (6)	C26—C15—C16—C17	3.1 (5)
C2—C3—C4—C5	−150.3 (3)	C15—C16—C17—C18	−149.2 (3)
C2—C3—C4—C9	31.8 (5)	C15—C16—C17—C22	33.4 (5)
C3—C4—C5—C6	179.8 (3)	C16—C17—C18—C19	−179.4 (3)
C9—C4—C5—C6	−2.1 (5)	C22—C17—C18—C19	−1.7 (4)
C3—C4—C9—C8	179.7 (3)	C16—C17—C22—C21	178.4 (3)
C5—C4—C9—C8	1.7 (5)	C18—C17—C22—C21	0.9 (4)
C4—C5—C6—C7	1.1 (5)	C17—C18—C19—C20	2.1 (5)
C5—C6—C7—C8	0.4 (5)	C18—C19—C20—C21	−1.6 (5)
C5—C6—C7—C10	179.4 (3)	C18—C19—C20—C23	177.2 (3)
C6—C7—C8—C9	−0.8 (5)	C19—C20—C21—C22	0.8 (5)
C10—C7—C8—C9	−179.8 (3)	C23—C20—C21—C22	−177.9 (3)
C6—C7—C10—C11	−111.1 (3)	C19—C20—C23—C24	50.7 (4)
C6—C7—C10—C12	123.4 (3)	C19—C20—C23—C25	−72.6 (4)
C8—C7—C10—C11	68.0 (4)	C21—C20—C23—C24	−130.6 (3)
C8—C7—C10—C12	−57.6 (4)	C21—C20—C23—C25	106.1 (4)
C7—C8—C9—C4	−0.3 (6)	C20—C21—C22—C17	−0.6 (5)

Symmetry codes: (i) *x*, *y*, *z*+1; (ii) *x*, *y*, *z*−1; (iii) −*x*+1, −*y*+2, −*z*; (iv) −*x*+1, −*y*+1, −*z*; (v) −*x*, −*y*+2, −*z*; (vi) *x*−1, *y*, *z*; (vii) −*x*+2, −*y*+1, −*z*+1; (viii) *x*+1, *y*, *z*.

### Hydrogen-bond geometry (Å, °)

**Table:**

*D*—H···*A*	*D*—H	H···*A*	*D*···*A*	*D*—H···*A*
O1—H1···O4^i^	0.94 (4)	1.71 (4)	2.644 (4)	175 (3)
O3—H3A···O2^ii^	0.93 (4)	1.71 (4)	2.631 (3)	169 (3)
C3—H3···O1	0.96 (3)	2.35 (2)	2.707 (4)	101.2 (16)
C13—H13A···O2	0.9600	2.2800	2.759 (4)	110.00
C16—H16···O3	0.91 (3)	2.31 (2)	2.698 (4)	105.1 (18)
C26—H26A···O4	0.9600	2.3000	2.770 (4)	110.00

Symmetry codes: (i) *x*, *y*, *z*+1; (ii) *x*, *y*, *z*−1.
